# Arsenic trioxide enhances the chemotherapeutic efficiency of cisplatin in cholangiocarcinoma cells via inhibiting the 14-3-3ε-mediated survival mechanism

**DOI:** 10.1038/s41420-020-00330-x

**Published:** 2020-09-21

**Authors:** Ming Jin, Liunan Wu, Shuai Chen, Rong Cai, Yi Dai, Haojun Yang, Liming Tang, Yuan Li

**Affiliations:** 1 The Collaborative Innovation Center for Cancer Personalized Medicine, School of Public Health, Nanjing Medical University, 211166 Nanjing, China; 2 Department of General Surgery, The Affiliated Changzhou No. 2 Hospital of Nanjing Medical University, 213003 Changzhou, China

**Keywords:** Cancer therapy, Chemotherapy

## Abstract

Cholangiocarcinoma (CCA) is the second most frequent primary liver carcinoma with high degrees of malignancy and mortality. Chemotherapy plays a key role in the treatment of CCA, however, the low chemotherapeutic efficiency leads to a bottleneck. So unraveling the potential mechanisms to enhance the efficiency (reduced the dosage and enhanced the effects of chemotherapy drugs) and identifying alternative therapeutic strategies in CCA are urgently needed. Here, we found that, in CCA cells, when cisplatin (CDDP) displayed anti-tumor effects, it activated 14-3-3ε simultaneously, which in turn formed a survival mechanism via the phosphorylation of phosphatidylinositol 3-kinase/protein kinase B (PI-3K/Akt). However, low concentrations of arsenic trioxide (ATO) could disrupt such survival mechanism and enhanced the efficiency. For the molecular mechanisms, ATO attenuated 14-3-3ε at both transcriptional and post-transcriptional (ubiquitination degradation) levels. Such repressive effect blocked the activation of PI-3K/Akt, and its downstream anti-apoptotic factors, B-cell lymphoma 2 (Bcl-2), and survivin. Collectively, our present study revealed that the synergistic effects of ATO and CDDP could be a novel approach for enhancing the efficiency, which provides an innovative therapeutic vision for the treatment of CCA.

## Introduction

Cholangiocarcinoma (CCA) is the second most frequent primary liver malignancy in humans after hepatocellular carcinoma (HCC) worldwide^1^. Over the past decades, both incidence and mortality of CCA had been increasing gradually^2^. Surgical operation is the best chance for patients with CCA for long-term survival, nevertheless, most of the patients lose the surgical indications due to the advanced-stage of detection; so chemotherapy is needed to provide benefits for the CCA^3^. However, the efficiency of chemotherapy is restricted partly due to: (1) abundant extracellular matrix and cancer-associated fibroblasts shape the specific tumor microenvironment, help cancer cells get rid of the cytostatic drugs, cisplatin; and (2) cisplatin has great cytotoxic and side effects^4,5^. Therefore, unraveling the potential mechanisms to enhance the efficiency of chemotherapy drugs (reduced the dosage and enhanced the effects of chemotherapy drugs) and identifying alternative therapeutic strategies in CCA are urgently needed.

Arsenic trioxide (ATO) has been in use in ancient traditional Chinese medicine to treat several diseases^6^, and has been recognized as the first-line therapeutic agent for acute promyelocytic leukemia^7^. Meanwhile, ATO is also used to suppressing the progression of many solid tumors, such as breast and liver^8^. We previous revealed that ATO could effectively attenuated the growth/angiogenesis, metastasis, self-renewal, and multidrug resistance via epigenetic modification, regulation of related molecules and chemokines in HCC cells^9–12^. Nevertheless, the roles of ATO in regulating chemotherapeutic efficiency of CCA, and the potential mechanisms involved in, remains uninvestigated.

The 14-3-3 proteins are a family of phosphoserine/threonine binding proteins of ~28 to 33 kDa acidic polypeptides, including seven highly homologous isoforms: β, γ, σ, ε, ζ, η, and θ^13^. Accumulative evidence indicated that 14-3-3 family was involved in regulating the growth, survival, metastasis, and showed potential beneficial effects of therapies in various cancers^14^. According to our previous researches, 14-3-3η isoform was a novel characteristic neoplastic factor in HCC, inducing the growth/angiogenesis and MDR properties^15,16^. Moreover, by inhibiting 14-3-3 η, ATO reversed the MDR in HCC cells^11^. However, it is still uninvestigated that whether there are isoforms of 14-3-3 family could function with ATO and enhance the chemotherapeutic efficiency in CCA. Here, to investigate the functions of ATO in regulating the chemotherapeutic efficiency of CCA, and to reveal the potential biological process, we treated the CCA cells with cisplatin in the presence or absence of ATO, and explored the underlying mechanisms with emphases on the CCA-related specific isoforms of 14-3-3.

## Results

### A low concentration of ATO enhanced the efficiency of CDDP in CCA cells

**Fig. 1:**
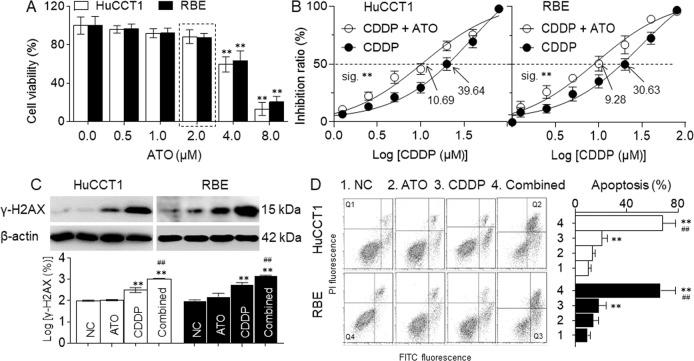
A low concentration of ATO enhanced the efficiency of CDDP in CCA cells. **a** HuCCT1 and RBE cells were treated with different concentrations (0.0 to 8.0 μM) of ATO for 24 h, and then the cell viabilities were determined in triplicate. **b** HuCCT1 and RBE cells were treated with different concentrations of CDDP (0.0 to 80 μM) combined with 0.0 or 2.0 μM ATO for 24 h, respectively. The cell viability was determined in triplicate, and the IC_50_s were calculated. **c** and **d** CCA cells treated by 10.0 μM CDDP in the presence or absence of 2.0 μM ATO for 24 h. **c** Western blot (top) and quantitative analysis (bottom) of the expressions of γ-H2AX. **d** Percentage of cell apoptosis (Q1: necrotic cells, Q2: late apoptosis, Q3: early apoptosis, Q4: living cells). The right graph was a statistical analysis of total apoptosis (Q2 + Q3). **p < 0.01 vs. control, ^##^ *p* < 0.01 vs. CDDP-treated group.

First, we treated the human intrahepatic biliary epithelial cells (HiBEC) were with ATO or CDDP, and found that, the IC_50_s (μM) for these two reagents were 7.528 and 17.38, respectively (Supplementary Fig. S1). For cancer chemotherapy, ATO was used with concentration of ~8.0 μM in the patients’ plasma^17,18^. Here, HuCCT1 and RBE cells were treated with different concentrations (0.0 to 8.0 μM) of ATO for 24 h, for cell viabilities, the no observed adverse effect level of ATO was ~2.0 μM (Fig. 1a). We then treated these two cells with different concentrations of CDDP (0.0 to 80 μM) combined with 0.0 or 2.0 μM ATO for 24 h. As shown in Fig. 1b, the IC_50_s (μM) of CDDP-treated alone group and CDDP combined with ATO treatment group were: 39.64 vs. 10.69 (HuCCT1) and 30.63 vs. 9.28 (RBE). As a cell cycle nonspecific drug, CDDP not only inhibited the cell proliferation, but also caused DNA damage and apoptosis. Here, HuCCT1 and RBE cells were treated by 10.0 μM CDDP in the presence or absence of 2.0 μM ATO for 24 h. Compared with CDDP-treated alone group, CDDP plus ATO treatment significantly enhanced the DNA damage (Fig. 1c) and apoptosis (Fig. 1d). Collectively, these results suggested that, a low concentration of ATO facilitated the CDDP-induced inhibition of cell proliferation, DNA damages, and apoptosis, enhancing the efficiency of CDDP in CCA cells.

### Identification of 14-3-3ε isoform as a CCA-associated 14-3-3 family member, attenuating the efficiency of CDDP in CCA cells

**Fig. 2:**
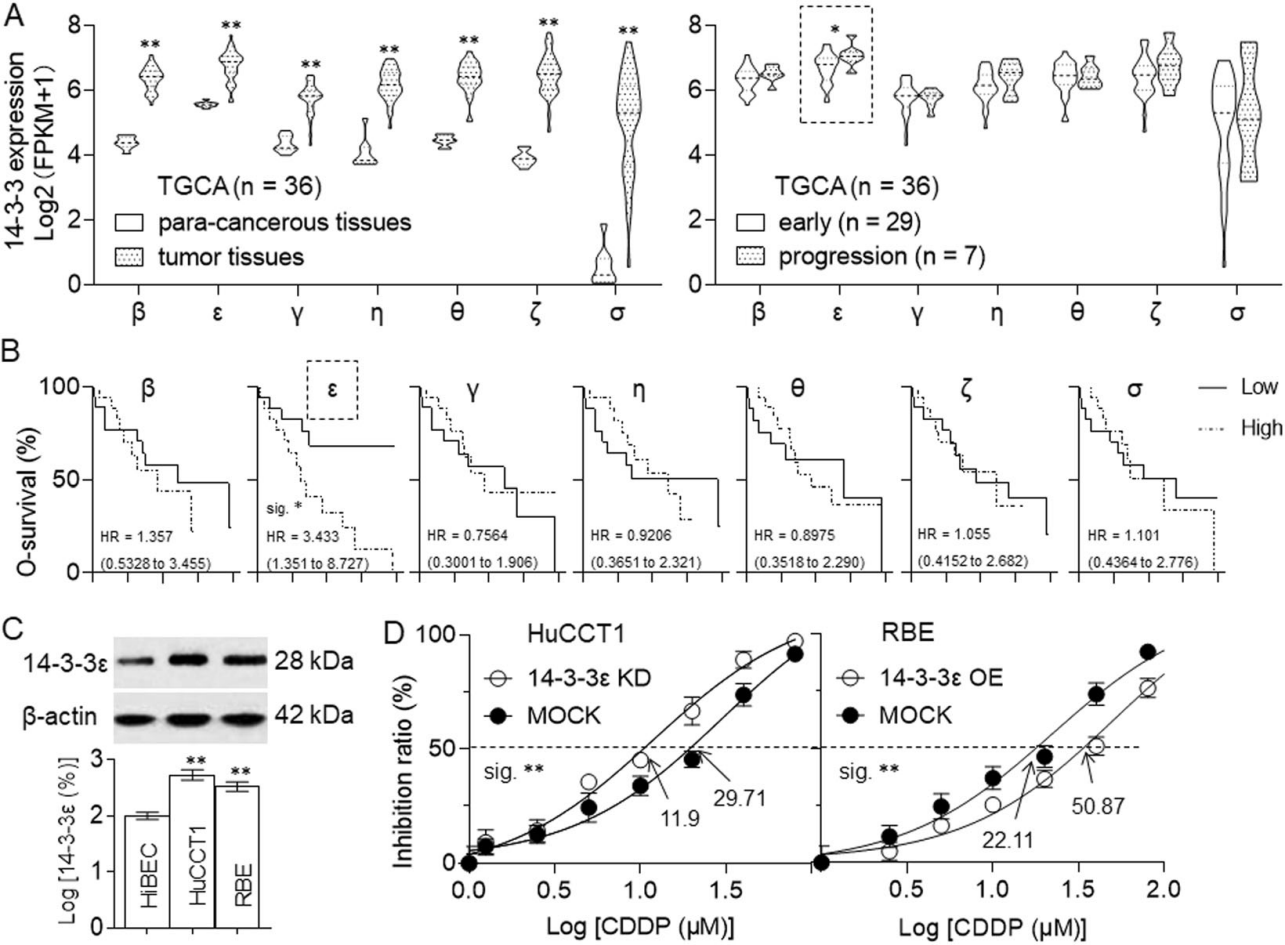
Identification of 14-3-3ε isoform as a CCA-associated 14-3-3 family member, attenuating the efficiency of CDDP in CCA cells. **a** Expression levels of 14-3-3 family in tumor tissues/para-cancerous tissues (left) or in early stage/progression of tumor (right) based on the UCSC Xena database (https://xena.ucsc.edu). **b** Prognostic significance of 14-3-3 family based on the UCSC Xena database. **c** Western blot (top) and quantitative analysis (bottom) of the expressions of 14-3-3ε in HIBEC, HuCCT1, and RBE cells. **d** HuCCT1 cells were transfected by NC- or 14–3-3ε-siRNA, while RBE cells were transfected by vector or 14-3-3ε-plasmid. After then, they were treated by different concentrations of CDDP for 24 h. The cell viability was determined in triplicate, and the IC_50_s were calculated. Note: KD knockdown, OE overexpression. **p < 0.01 vs. HiBEC or MOCK group.

Based on the TCGA database, all the seven isoforms were higher expressed in CCA than those in para-cancerous tissues; however, only the expression of 14-3-3ε was significantly associated with the tumor progression (Fig. 2a). Further, 14-3-3ε was also the only isoform, predicting the worse survival (Fig. 2b). Increasing evidence suggested that 14-3-3ε isoform had become critical in cell biology due to its functions in carcinogenesis and tumor progression^19,20^. Especially, in hepatocellular carcinoma, 14-3-3ɛ had an irreplaceable tumor-promoting role^21,22^. Here, compared with a normal bile duct epithelial cell line, HIBEC, the expression of 14-3-3ε was markedly higher in CCA, especially in HuCCT1 cells (Fig. 2c). Then we further knocked down the seven isoforms of 14-3-3 family in HuCCT1 cells independently, and found that knockdown of 14-3-3ε significantly decreased the IC_50_ of CDDP among the seven isoforms (Supplementary Table. S3); however, forced expression of 14-3-3ε in RBE cells exhibited the opposite phenomenon (Supplementary Fig. S2 and Fig. 2d). These results indicated that 14-3-3ε isoform was a CCA-associated 14-3-3 member regulating the efficiency of CDDP in CCA cells.

### Identification of PI-3K/Akt as an important phosphorylated signal pathway regulated by 14-3-3ε in CCA cells

**Fig. 3:**
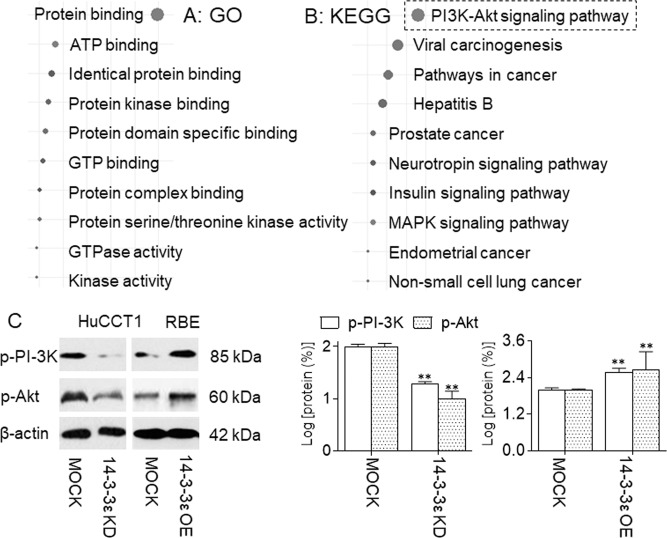
Identification of PI-3K/Akt as an important phosphorylated signal pathway regulated by 14-3-3ε in CCA cells. **a** and **b** GO biological process and KEGG pathway analyses of the 50 most frequently altered neighbor factors around 14-3-3ε from STRING database, the size of the dot represents the number of genes enriched in the item. **c** HuCCT1 cells were transfected by NC- or 14–3-3ε-siRNA, while RBE cells were transfected by vector or 14-3-3ε-plasmid. Western blot (left) and quantitative analysis (right) of the expressions of p-PI-3K/p85 subunit and p-Akt. **p < 0.01 vs. MOCK group.

To further predict the 14-3-3ε-regulated potential downstream signal transduction mechanisms, a search tool for the retrieval of interacting genes (STRING) database were employed. We took 14-3-3ε as the central molecule and constructed a protein–protein interaction via the generation of 50 most frequently altered neighbor factors around it (Supplementary Fig. S3). Next, the database for annotation, visualization and integrated discovery (DAVID) was employed to conduct GO and KEGG pathway analysis based on the above-mentioned 50 factors. Corresponded to the classic phosphoserine/threonine binding/regulating functions of 14-3-3 family^13,23^, the protein binding, protein kinase binding, and protein serine/threonine kinase activity, etc. were identified as the top 10 molecular functions (Fig. 3a). Then, via the KEGG pathway enrichment, we further identified the top 10 pathways related to the functions of 14-3-3ε, and found that, the PI-3K/Akt pathway was ranked the first among the pathways involved in (Fig. 3b). Verifying the bioinformatics results, we transfected NC-, 14-3-3ε-small interfering RNA (siRNA), vector, or 14-3-3ε-plasmid in CCA cells. As shown in Fig. 3c and Supplementary Fig. S4, knockdown of 14-3-3ε attenuated the phosphorylation of PI-3K/p85 subunit and Akt, while forced expression of 14-3-3ε showed the opposite effects. As PI-3K/Akt played a critical role in the response to chemotherapy^24,25^, we hypothesized that the 14-3-3ε/PI-3K/Akt was involved in regulating the chemotherapy efficiency in CCA.

### Effects of CDDP and/or ATO on 14-3-3ε/PI-3K/Akt signaling in CCA cells

**Fig. 4:**
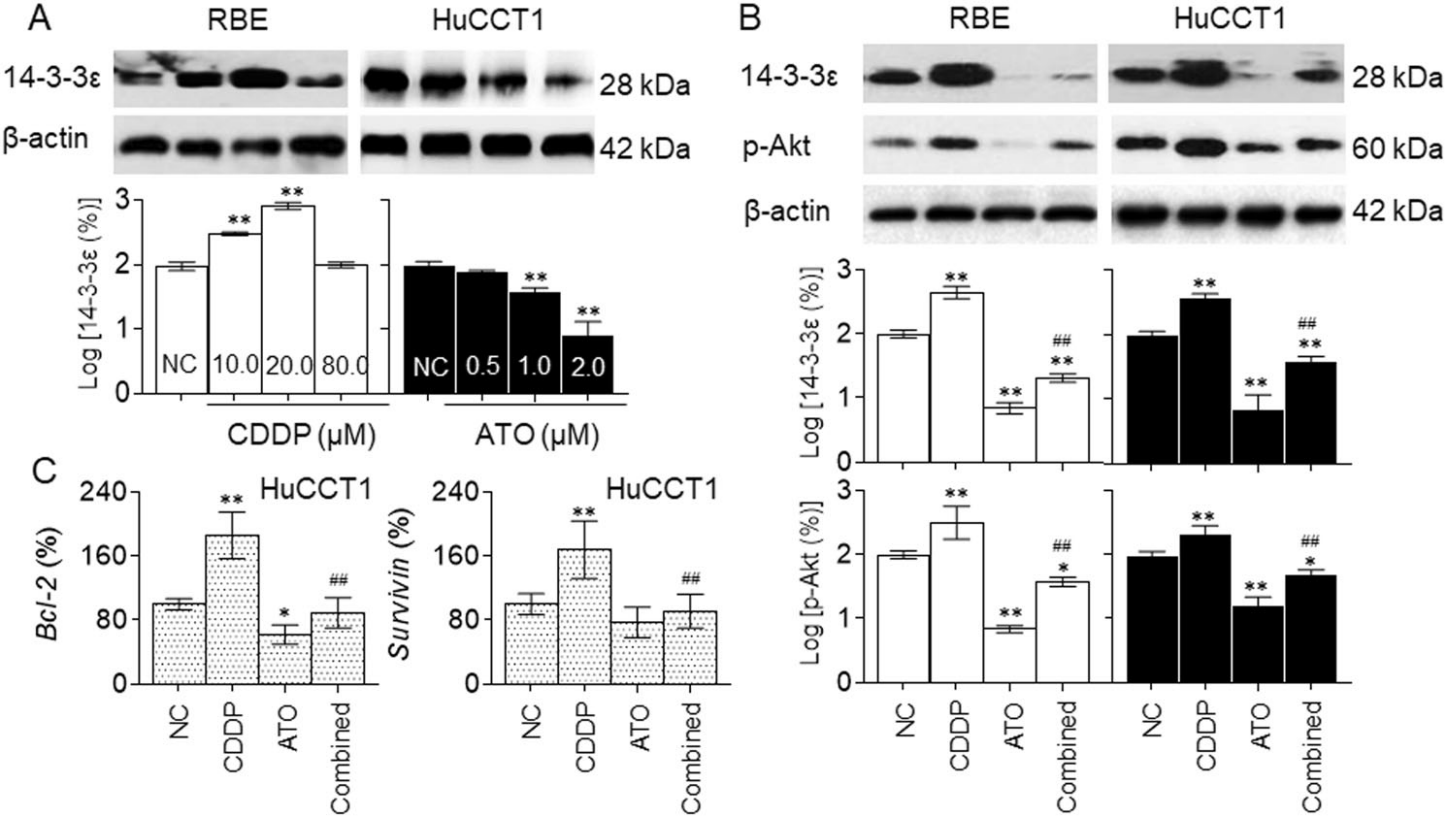
Effects of CDDP and/or ATO on 14-3-3ε/PI-3K/Akt signaling in CCA cells. **a** CCA cells were treated with 0.0, 10.0, 20.0, or 80.0 μM of CDDP or with 0.0 to 2.0 μM of ATO for 24 h. Western blot (top) and quantitative analysis (bottom) of the expressions of 14-3-3ε. **b** and **c** HuCCT1 or RBE cells were treated with 10.0 μM of CDDP in the presence or absence of 2.0 μM of ATO for 12 (to determine the mRNAs) or 24 h (to determine the proteins). **b** Western blot (top) and quantitative analysis (bottom) of the expressions of 14-3-3ε or p-Akt. **c** qPCR analysis in triplicate of the *Bcl-2* and *survivin* mRNAs. *p < 0.05 and **p < 0.01 vs. control group; ^##^*p* < 0.01 vs. CDDP-treated group.

We then investigated the effects of CDDP and/or ATO on 14-3-3ε/PI-3K/Akt signaling. HuCCT1 or RBE cells were firstly exposed to different concentrations of CDDP or ATO, respectively. Within a relatively low concentration range of CDDP (0.0 to 20.0 μM), the expression of 14-3-3ε was elevated in a dose-dependent manner, while in high-dose group (80.0 μM), the phenomenon was not observed. On the contrary, low concentrations of ATO (0.0 to 2.0 μM) inhibited the expression of 14-3-3ε dose-dependently (Fig. 4a). Next, we treated HuCCT1 or RBE cells with 10 μM CDDP in the presence or absence of 2.0 μM ATO, as shown in Fig. 4b, c, ATO markedly attenuated the CDDP-induced increased expression of 14-3-3ε, which in turn inhibited the CDDP-induced activation of Akt and its downstream anti-apoptotic factors, Bcl-2 and survivin. Previous studies revealed that CDDP in the relatively low concentrations could upregulate phosphorylation levels of p-Akt while high-concentration inhibit it^26,27^. Specifically, PI-3K/Akt is a key signaling pathway of particular relevance to anti-apoptosis and survival in cancer progressions^28,29^. So, our present data suggested that, in CCA cells, when a relative lower concentration of CDDP caused DNA damages and apoptosis, the CDDP also activated 14-3-3ε/PI-3K/Akt signaling pathway simultaneously, which in turn formed a survival mechanism. However, low concentrations of ATO could disrupt such survival mechanism, and then combine with CDDP to exert the functions of enhancing efficiency on chemotherapy of CCA.

### The inhibition of 14-3-3ε was involved in the ATO-enhanced chemotherapy efficiency of CDDP in CCA cells

**Fig. 5:**
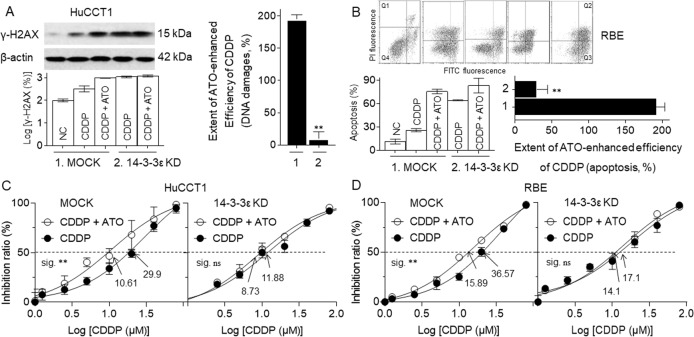
The inhibition of 14-3-3ε was involved in the ATO-enhanced chemotherapy efficiency of CDDP in CCA cells. After HuCCT1 or RBE cells were transfected by NC- or 14–3-3ε-siRNA, they were treated with 10.0 μM of CDDP in the presence or absence of 2.0 μM of ATO. **a** Western blot (left, top) and quantitative analysis (left, bottom) of the expressions of γ-H2AX in HuCCT1 cells. The right graph was a statistical analysis of the extent of ATO-enhanced efficiency of CDDP (DNA damages, %). (**b**, top, and bottom, left) Percentage of cell apoptosis (Q2 + Q3). The right graph was a statistical analysis of the extent of ATO-enhanced efficiency of CDDP (apoptosis, %). **c** and **d** The cell viability was determined in triplicate, and the IC_50_s were calculated. **p < 0.01 vs. MOCK group; ^##^*p* < 0.01 vs. CDDP-treated group.

Based on the above-mentioned results, we speculated that, the inhibition of 14-3-3εby ATO was a critical biological progression, which was involved in the ATO-enhanced chemotherapy efficiency of CDDP in CCA cells. To confirm this speculation, 14-3-3ε was knocked down in CCA cells. Here, in NC-siRNA transfected HuCCT1 cells, compared with CDDP-treated alone group, CDDP plus ATO treatment significantly enhanced the DNA damage; however, in 14-3-3ε-siRNA transfected cells, CDDP treated alone could induce the remarkable DNA damage, while ATO combined with CDDP only caused a slightly enhancement (Fig. 5a). The similar phenomenon was displayed in the degrees of apoptosis in RBE cells (Fig. 5b). Furthermore, in NC-siRNA transfected cells, the IC_50_s (μM) of CDDP-treated alone group and CDDP combined with ATO treatment group were 29.9 vs. 10.61 (HuCCT1) and 36.57 vs. 15.89 (RBE), however, in 14-3-3ε-siRNA transfected cells, the IC_50_s (μM) for the above-mentioned two groups were 11.88 vs. 8.73 (HuCCT1) and 17.1 vs. 14.1 (RBE, Fig. 5c, d). These results indicated that the inhibition of 14-3-3ε played an important role in the ATO-enhanced chemotherapy efficiency of CDDP in CCA cells.

### Potential mechanisms underlying the ATO-induced inhibition of 14-3-3ε

**Fig. 6:**
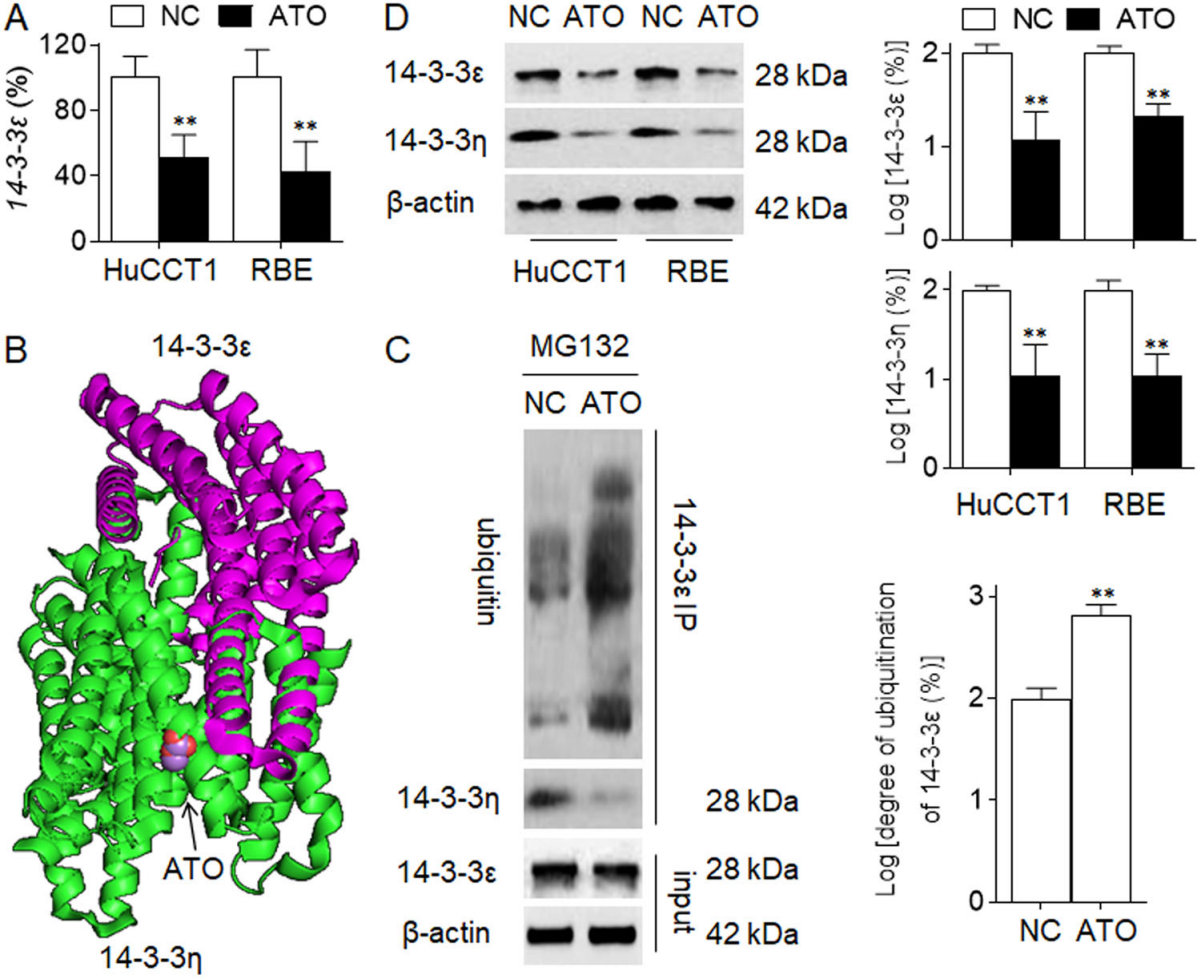
Potential mechanisms underlying the ATO-induced inhibition of 14-3-3ε. **a** HuCCT1 and RBE cells were treated by 0.0 or 2.0 μM of ATO for 12 h, qPCR analysis in triplicate of *14-3-3ε* mRNAs. **b** PyMol software analyses of the relationship between 14-3-3ε and14-3-3η, and the binding of ATO to this heterodimers. **c**, left After HuCCT1 cells were pre-treated by 20 μM of MG-132 for 2 h, they were treated by 0.0 or 2.0 μM of ATO for 6 h, Co-IP analyses of the binding of 14-3-3ε with 14-3-3η or ubiquitin. **c**, right The graph was a statistical analysis for the ubiquitination degree of 14-3-3ε. **d** HuCCT1 and RBE cells were treated by 0.0 or 2.0 μM of ATO for 24 h, Western blot (left) and quantitative analysis (right) of the expressions of 14-3-3ε and 14-3-3η. **p < 0.01 vs. control group.

First, we found that ATO significantly decreased the expression of *14-3-3ε* mRNA in CCA cells (Fig. 6a), suggesting a transcriptional inhibition. Previous study revealed that, PI-3K/Akt could transcriptional activated 14-3-3ε^30^. Here, we found that 14-3-3ε upregulated PI-3K/Akt. So, we speculated that ATO transcriptional blocked *14-3-3ε* via disrupting the 14-3-3ε/PI-3K/Akt feedback loop. Then we further investigated if there was a post-transcriptional regulation effect of ATO on 14-3-3ε. Previous studies indicated that several isoforms of 14-3-3 family members had the abilities to form heterodimers, while 14-3-3ε isoform preferentially formed heterodimers among them particularly, and it heterodimerized readily with 14-3-3η^31,32^. Our previous studies found that, ATO targeted 14-3-3η for ubiquitination and degradation^11^. So, we conjectured that, via targeting 14-3-3η, ATO altered the 14-3-3η/14-3-3ε heterodimers, accelerating the degradation of 14-3-3ε (Fig. 6b). Verifying this hypothesis, we pre-treated HuCCT1 cells with MG-132 (a proteasome inhibitor), and found that, ATO treatment broke the 14-3-3η/14-3-3ε heterodimers, and enhanced the ubiquitination of 14-3-3ε (Fig. 6c), leading to attenuating the protein levels of 14-3-3η and 14-3-3ε in HuCCT1 and RBE cells (Fig. 6d). These results indicated that, ATO inhibited the 14-3-3ε at both transcriptional and post-transcriptional, might via blocking 14-3-3ε/PI-3K/Akt feedback loop and inducing the ubiquitination and degradation of 14-3-3ε in CCA cells.

## Discussion

Traditional Chinese medicine ATO is the first-line treatment of acute promyelocytic leukemia, and has also been approved for the treatment of human primary HCC by the State Food and Drug Administration of China^33,34^. Previous study showed that, acute promyelocytic leukemia patients needed to take 0.16 mg/kg/day ATO, and the treatment process often within a period of 6 weeks, the drug concentration level of ATO in plasma drug usually reached ~8 μM^17^. Although ATO contribute to improve the prognosis of the diseases to some extent, in clinical treatment, compared with the hematological diseases, the anti-cancer activity of solid tumors requires higher dose of ATO, which is often accompanied by severe toxicity and various adverse reactions, like fatigue, anorexia, vomiting, diarrhea, aspartate transaminase, hyponatremia, hypokalemia and so on^35^. Therefore, current researches are mainly focused on the combination therapeutic regimen instead of attempting single drug treatment merely. In present study, we found that low-dose ATO (concentrations far lower than clinical application) combined with cisplatin effectively increased the drug efficiency in CCA cell lines. This synergistic effect of ATO provides an innovative therapeutic vision for new applications of old drugs.

The 14-3-3 proteins had been widely reported as central regulators in cell cycle, growth, differentiation, apoptosis and migration, and thus participated in occurrence and development of tumors^14^. Studies suggested that several isoforms of 14-3-3 were found to be involved in hepatobiliary cancer, while different isoforms were implicated in the oncogenesis and development of cancers via diverse mechanisms. Among them, 14-3-3β, 14-3-3γ, 14-3-3ε, 14-3-3ζ, and 14-3-3η isoforms were reported to overexpress in CCA or HCC, implying their roles in promoting tumors^23,36^. Furthermore, these five members were also involved in the cell proliferation, tumor growth, metastasis and chemotherapy resistance in CCA or HCC^21,37,38^. Here, findings based on the TCGA database surprisingly indicated that, in addition to the high expression of 14-3-3ε in CCA, its expression trend was consistent with the development process of tumor, and even had merits of prognosis. Increasing evidence suggested that 14-3-3ε isoform had become critical in cell biology due to its functions in carcinogenesis and tumor progression^19^. Previous studies showed that the role 14-3-3ɛ played in the progression of tumors remains under contention. It has been identified that 14-3-3ɛ functioned as an oncogene in several types of tumors, such as papillary thyroid, meningioma and some others^39,40^. 14-3-3ɛ had an irreplaceable role in HCC, for mechanisms, it contributed to promote cell migration via the activation of the NF-κB/FAK pathway and also stimulate EMT in HCC by suppressing E-cadherin via Zeb-1^21,22^. As for gastric cancer, 14-3-3ɛ might participate in the expression of cyclins binding to CDKs, and then degrading phosphorylated p27^kip1^ to regulate the proliferation and progression of gastric cancer^20^. Thus, phosphorylation regulation brings into play the essential role in the function of 14-3-3ɛ.

CDDP is an inducer of reactive oxygen species (ROS) and ROS is a crucial mechanism for CDDP to induce cytotoxicity^41^. Studies revealed that, when CDDP caused the production of a certain range of ROS, which lead to oxidative stress, cellular adaptions might occur, survival-promoting signaling was also elicited by an increase in the ROS steady state level^42^. Previous studies reported, ROS in steady state is helpful to maintain the redox balance of cells, while the increased ROS production and lack of protective mechanism may cause elevated oxidative stress levels, which may lead to toxicity due to the interference of signal transduction pathways^43^. Here, we discovered that while CDDP showed anti-tumor effects via DNA damages and apoptosis, also activated 14-3-3ε/PI-3K/Akt signaling pathway and forming a survival mechanism, while high-dose cisplatin could not activate the above protective mechanism even result in severe adverse effects, which might be related to the antioxidant system. The results consistent with prior studies that have reported that CDDP in the relatively low concentrations could upregulate phosphorylation levels of p-Akt while high-concentration inhibit it^26,27^. Specifically, PI-3K/Akt is a key signaling pathway of particular relevance to the role of redox balance in cancer progressions. Mechanically, ROS mediated hyperphosphorylation of PI-3K and activated Akt as well as several anti-apoptotic factors. In addition, excessive oxidative stress activated the PI-3K/Akt through inhibiting the activity of PTEN(negative regulator), ROS at a high level may increase its stability and disturb the normal functions via modulating the phosphorylation^44^. Moreover, aberrant PI-3K/Akt signaling could also contribute to various molecular mechanisms to cause high ROS levels via activating NADPH oxidase^45^. Here, we found that low concentrations of ATO could intervene the survival mechanism by inhibiting 14-3-3ε/PI-3K/Akt, then we hypothesized that oxidative stress might play an irreplaceable role in the functions of enhancing efficiency on chemotherapy of CCA of combined treatment of CDDP and ATO.

## Conclusions

**Fig. 7:**
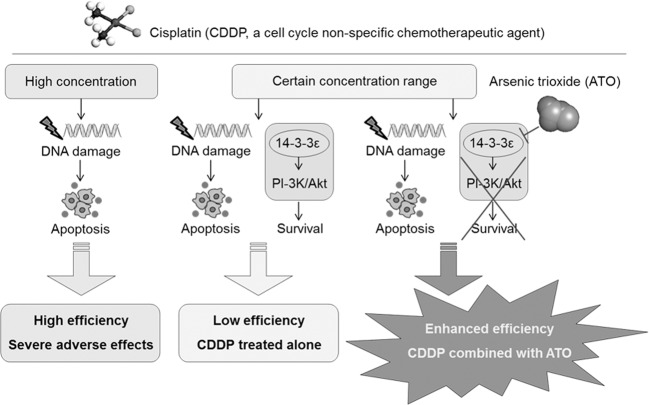
A sketch map summarizing the conclusion and potential clinical significance of our present study. ATO enhances the chemotherapeutic efficiency of CDDP in CCA cells via inhibiting the 14-3-3ε-mediated survival mechanism.

As a cell cycle nonspecific drug, high-concentrations of CDDP induced significant DNA damages and apoptosis, and could not activate the survival mechanism; nevertheless, it caused great toxicity and side effects (Fig. 7, left). A relative low concentration range of CDDP not only exerted the anti-tumor effect, but also elevated 14-3-3ε, the only survival-related 14-3-3 family member in CCA. Via the phosphine/threonine regulating function, 14-3-3ε activated PI-3K/Akt signal pathway, forming a survival mechanism to reduce the efficiency of CDDP (Fig. 7, middle). Importantly, via regulating 14-3-3ε at transcriptional and post-transcriptional levels, low concentrations of ATO attenuated 14-3-3ε, blocked the activation of PI-3K/Akt, broke the CDDP-induced survival mechanism, and finally enhanced the efficiency of CDDP (Fig. 7, right).

## Materials and methods

### Cell culture and reagents

The human CCA cell line, HuCCT1 was obtained from Institute of Biochemistry and Cell Biology, Chinese Academy of Science (Shanghai, China), while RBE was obtained and short tandem repeat identified by KeyGen Co. Ltd (Nanjing, China). Cells were cultured in RPMI-1640 medium (Gibco, Grand Island, NY), supplemented with 10% fetal bovine serum (FBS), 100 U/ml penicillin, 100 μg/ml streptomycin (Gibco), and maintained in a humidified incubator containing 5% CO_2_ at 37 °C. A mycoplasma stain assay kit (Beyotime, Haimen, China) was used for mycoplasma testing to rule out the possibility of cryptic contamination. Arsenic trioxide (ATO, > 99.0% purity) and cisplatin (CDDP ≥ 99% purity) were purchased from Sigma-Aldrich Co. Ltd (Shanghai, China).

### Cell viabilities and calculation of the 50% inhibitory concentrations (IC_50_)

In 96-well plates, a total of 2 × 10^3^ HuCCT1 or RBE cells were seeded and treated as indicated for 24 h, and their viabilities were determined by incubating with 10.0 μl of CCK-8 solution (Beyotime) for another 4 h. The absorbance at 450 nm was measured with a multi-well plate reader (Bio-Rad, CA, USA). The cell viability was determined in triplicate. A three-parameter dose–response equation and calculated with a non-linear regression was used to calculated the inhibition ratio (based on the data from measured absorbance), and a sigmoidal curve was generated via a graph-pad 8.0 software (CA, USA) to acquire the IC_50_ value. The results were displayed as “best fit values” ± “standard errors”^11^.

### Determination of cell apoptosis by flow cytometry

After HuCCT1 or RBE cells were treated as indicated for 24 h, they were harvested with trypsin-EDTA, washed twice with phosphate buffered saline by centrifugation and fixed with 1 ml of ice-cold 70% ethanol overnight. As we described previously^16^, the fixed cells were centrifuged, suspended in lysis buffer and incubated with RNase A for 10 min at room temperature. Cell apoptosis analyses were performed using Annexin V-FITC and propidium iodide kit (Beyotime) according to the manufacturer’s instruction, followed by flow cytometry analysis.

### Data mining and biological functional analysis

The original data (expression and clinical significance of 14-3-3 family members in CCA patients) was obtained from the Cancer Genome Atlas (TCGA) dataset via University of California Santa Cruz Xena browser (https://xenabrowser.net). The interactions between protein–protein or protein–chemicals were predicted via using a molecular docking software (The PyMOL Molecular Graphics System). A search tool for the retrieval of interacting genes (STRING) database (https://string-db.org) was employed to construct the protein–protein interaction. The database for annotation, visualization and integrated discovery (DAVID, http://david.ncifcrf.gov) was employed to process the gene ontology (GO) and kyoto encyclopedia of genes and genomes (KEGG) pathway analysis. The *p*-value < 0.05 was set as the cutoff criterion for the significant enrichment.

### Cell transfection

Scrambled and pcDNA-3.1-14-3-3ε-flag plasmid were synthesized by Generay Biotech Co. Ltd (Shanghai, China), while the NC- and 14-3-3ε-siRNA were purchased from Santa Cruz Biotechnology (http://datasheets.scbt.com/sc-29588.pdf). As we described previously, 5 ng/ml of plasmids or 20 nM of siRNAs were mixed with lipofectamine 3000 reagent (Invitrogen, Carlsbad, USA) in mediums containing 10% FBS without antibiotics; after transfection, such cells were cultured in fresh mediums supplemented with 10% FBS for another 24 h before being used for other experiments^11^.

### Quantitative real-time polymerase chain reaction (qRT-PCR)

As we described previously, the total RNA was isolated by Trizol (Invitrogen) and was reverse transcribed into complementary DNA (cDNA). qRT-PCR was performed in triplicate with Light Cycler 96 SYBR Green I Master Mix (Roche) machine and SYBR Green Master Mix (Vazyme Biotech Co, Ltd.). The primers used were listed in Supplementary Table S1. The β-actin was amplified to ensure cDNA integrity and to normalize expression. The fold changes in expression of each gene were calculated by a comparative threshold cycle (Ct) method using the formula 2^-(ΔΔCt)^ ^16^.

### Western blot

Total protein was extracted, then the concentrations were measured with the BCA kit (Beyotime). SDS-polyacrylamide gel electrophoresis followed by transferring the protein to polyvinylidene fluoride membranes were all according to our previous study^46^. The primary and secondary antibodies used were listed in Supplementary Table S2. The immune complexes were detected by enhanced chemiluminescence kit (Cell Signaling Technology). Densitometric analysis was determined in triplicate via the Image-pro-plus 6.0 software (Media Cybernetics, Georgia, USA).

### Co-immunoprecipitation (Co-IP)

The antibodies used were also listed in Supplementary Table S2. As we described previously^11^, cells were extracted for 30 min with immunoprecipitation lysis buffer (Beyotime). After centrifugation of the preparations, the concentrations of supernatants were measured with the BCA kit. Then 100 μg proteins were incubated with 14-3-3ε antibody at 4 ˚C overnight. Then the protein-antibody complex were incubated with IgA plus IgG sepharose beads (Beyotime) at 4 ˚C for another 12 h. After then, the supernatants were removed and the beads were washed for three times, resuspended in the SDS sample buffer (Beyotime), and boiled to remove protein from the beads. Then, such protein samples were analyzed by western blots.

### Statistical analysis

Data were presented as the means ± SD, and compared via a graph-pad 8.0 software. The biological functional analysis was performed by R language. The differences were analyzed by using Student’s *t*-test, one-way analysis of variance (ANOVA) followed by Dunnett’s *t-*test, or two-way analysis of variance followed by Sidak’s multiple comparisons test. *p* < 0.05 were considered statistically significant.

## Supplementary information

Table. S1. Primers used in this study

Table. S2. Antibodies used in this study.

Table. S3. Effects of 14-3-3 knockdown on the IC50s of CDDP in HuCCT1 cells

Fig. S1. The effects of ATO and CDDP on HiBEC cells.

Fig. S2. Knockdown or overexpression efficiency in HuCCT1 or RBE cells

Fig. S3. Fifty most frequently altered neighbor factors around 14-3-3ε

Fig. S4. Effects of 14-3-3ε on PI-3K/Akt in HuCCT1 or RBE cells

Supplementary Figure Legends
