# Supplementary material for: Arsenic trioxide enhances the chemotherapeutic efficiency of cisplatin in cholangiocarcinoma cells via inhibiting the 14-3-3ε-mediated survival mechanism
Source: Cell Death Discov. 2020 Sep 21;6:92. doi: 10.1038/s41420-020-00330-x (PMC7505839; doi:10.1038/s41420-020-00330-x)
Supplement: Supplementary file 1 — Table. S1. Primers used in this study [file 41420_2020_330_MOESM1_ESM.docx]

**Table. S1. Primers used in this study**

| Genes | Primers |
| --- | --- |
| *14–3-3ε* | F: 5'-TGCAGAACTGGATACGCTGAGTGA-3'  R: 5'- TCACCCTGCATGTCTGAAGTCCAT-3' |
| *Bcl-2* | F: 5’-TCCCTCGCTGCACAAATACTC-3’  R: 5’-TTCTGCCCCTGCCAAATCT-3’ |
| *Survivin* | F: 5’-AAGGCAGTGGCCTAAATCCT-3’  R: 5’-AGCCCGGATGATACAAACAG-3’ |
