# Supplementary material for: Arsenic trioxide enhances the chemotherapeutic efficiency of cisplatin in cholangiocarcinoma cells via inhibiting the 14-3-3ε-mediated survival mechanism
Source: Cell Death Discov. 2020 Sep 21;6:92. doi: 10.1038/s41420-020-00330-x (PMC7505839; doi:10.1038/s41420-020-00330-x)
Supplement: Supplementary file 2 — Table. S2. Antibodies used in this study. [file 41420_2020_330_MOESM2_ESM.docx]

**Table. S2. Antibodies used in this study.**

| Antibodies | Web Link | Used |
| --- | --- | --- |
| 14-3-3ε | https://www.cst-c.com.cn/datasheet.jsp?productId=9635&images=1&protocol=0 | 1: 1000 (WB)  1: 100 (IP) |
| 14-3-3η | https://www.cst-c.com.cn/datasheet.jsp?productId=9640&images=1&protocol=0 | 1: 1000 (WB) |
| p-PI-3K/p85 | https://www.cst-c.com.cn/datasheet.jsp?productId=17366&images=1&protocol=0 | 1: 1000 (WB) |
| p-Akt | https://www.cst-c.com.cn/datasheet.jsp?productId=4060&images=1&protocol=0 | 1: 1000 (WB) |
| Flag | https://www.beyotime.com/Manual/AF519%20Flag%E6%8A%97%E4%BD%93.pdf | 1: 1000 (WB) |
| ubiquitin | https://www.cst-c.com.cn/datasheet.jsp?productId=3933&images=1&protocol=0 | 1: 500 (WB) |
| β-Actin | https://www.beyotime.com/Manual/AA128%20Actin%E6%8A%97%E4%BD%93.pdf | 1: 1000 (WB) |
