# Supplementary material for: Arsenic trioxide enhances the chemotherapeutic efficiency of cisplatin in cholangiocarcinoma cells via inhibiting the 14-3-3ε-mediated survival mechanism
Source: Cell Death Discov. 2020 Sep 21;6:92. doi: 10.1038/s41420-020-00330-x (PMC7505839; doi:10.1038/s41420-020-00330-x)
Supplement: Supplementary file 3 — Table. S3. Effects of 14-3-3 knockdown on the IC50s of CDDP in HuCCT1 cells [file 41420_2020_330_MOESM3_ESM.docx]

**Table. S3. Effects of 14-3-3 knockdown on the IC_50_s of CDDP in HuCCT1 cells**

| Knockdown | IC_50_s (mean ± SE.) | *p*-value (*vs*. Mock) |
| --- | --- | --- |
| *Mock* | 29.71 ± 1.23 | */* |
| *14-3-3β* | 19.55 ± 1.19 | 0.3876 |
| *14-3-3ε* | 11.90 ± 1.17 | 0.0026 |
| *14-3-3γ* | 23.43 ± 1.26 | 0.4806 |
| *14-3-3η* | 13.83 ± 1.19 | 0.0497 |
| *14-3-3θ* | 14.48 ± 1.22 | 0.0901 |
| *14-3-3ζ* | 21.54 ± 1.23 | 0.8245 |
| *14-3-3σ* | 18.26 ± 1.22 | 0.5604 |
