# Supplementary figures and images for: Arsenic trioxide enhances the chemotherapeutic efficiency of cisplatin in cholangiocarcinoma cells via inhibiting the 14-3-3ε-mediated survival mechanism
Source: Cell Death Discov. 2020 Sep 21;6:92. doi: 10.1038/s41420-020-00330-x (PMC7505839; doi:10.1038/s41420-020-00330-x)

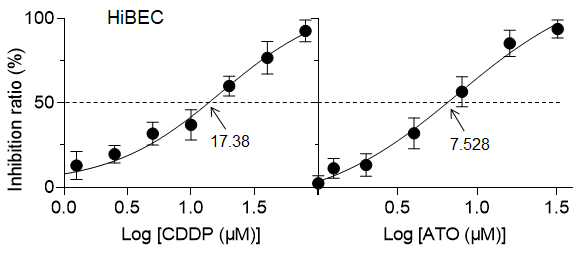

Supplement: Supplementary file 4 — Fig. S1. The effects of ATO and CDDP on HiBEC cells. [file 41420_2020_330_MOESM4_ESM.tif]

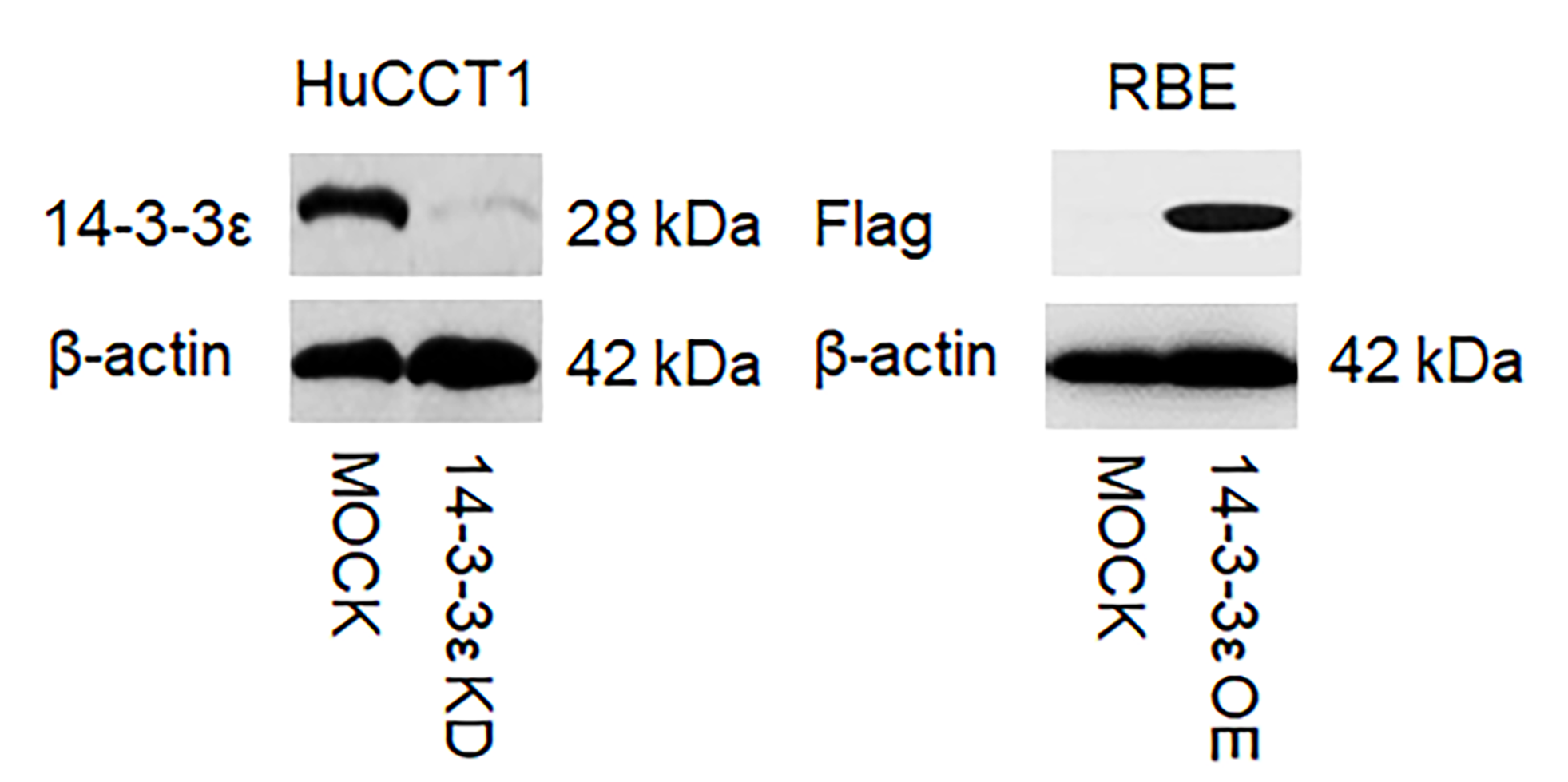

Supplement: Supplementary file 5 — Fig. S2. Knockdown or overexpression efficiency in HuCCT1 or RBE cells [file 41420_2020_330_MOESM5_ESM.tif]

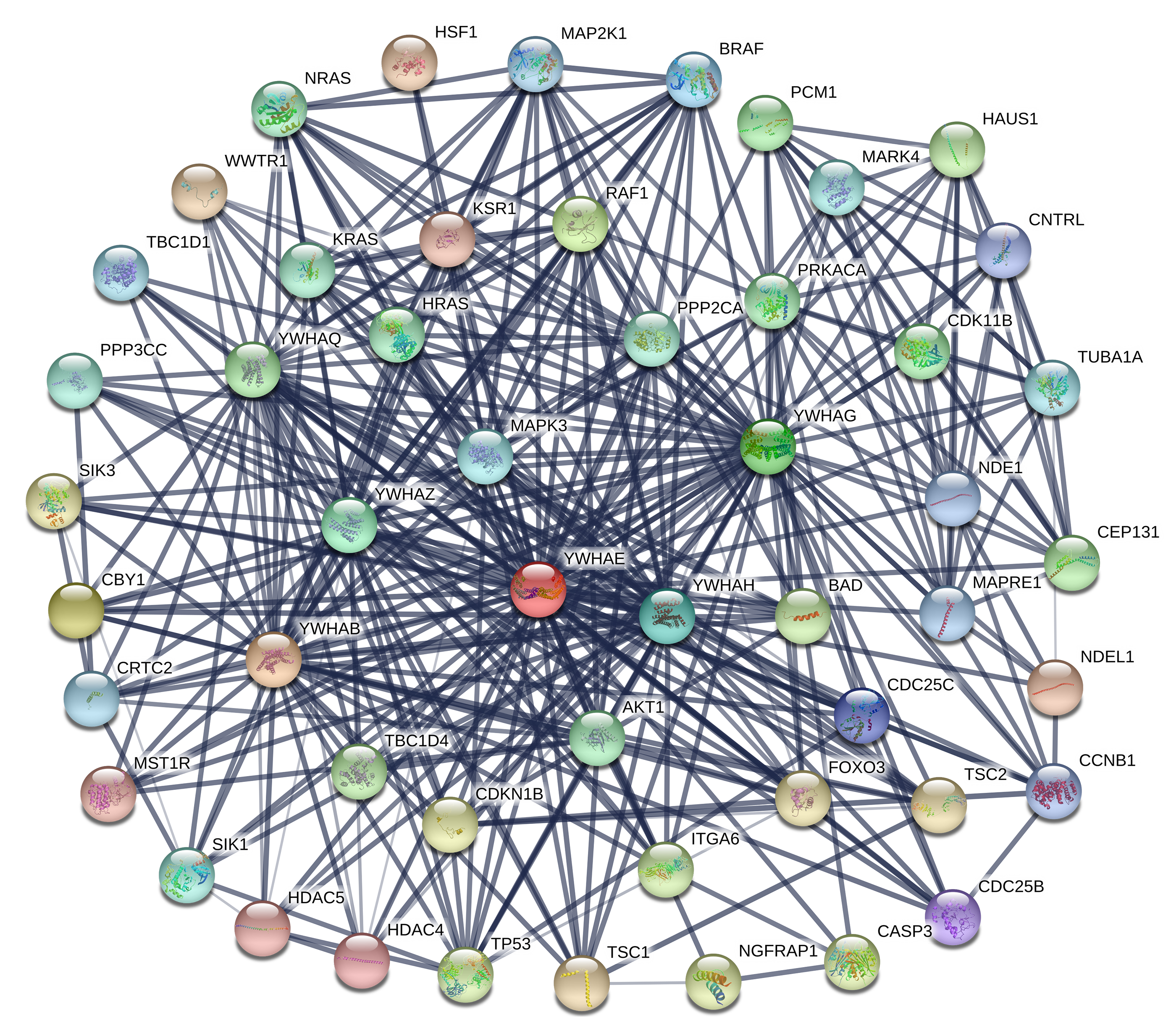

Supplement: Supplementary file 6 — Fig. S3. Fifty most frequently altered neighbor factors around 14-3-3ε [file 41420_2020_330_MOESM6_ESM.tif]

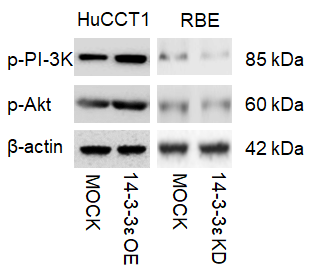

Supplement: Supplementary file 7 — Fig. S4. Effects of 14-3-3ε on PI-3K/Akt in HuCCT1 or RBE cells [file 41420_2020_330_MOESM7_ESM.tif]
