# Supplementary material for: Arsenic trioxide enhances the chemotherapeutic efficiency of cisplatin in cholangiocarcinoma cells via inhibiting the 14-3-3ε-mediated survival mechanism
Source: Cell Death Discov. 2020 Sep 21;6:92. doi: 10.1038/s41420-020-00330-x (PMC7505839; doi:10.1038/s41420-020-00330-x)
Supplement: Supplementary file 8 — Supplementary Figure Legends [file 41420_2020_330_MOESM8_ESM.docx]

**Supplementary Figure Legends**

**Fig. S1. The effects of ATO and CDDP on HiBEC cells.**

HiBEC cells were treated with different concentrations of CDDP (0.0 to 80 μM) or ATO (0.0 to 32.0 μM) for 24 h, the cell viabilities were determined in triplicate, and the IC_50_s were calculated.

**Fig. S2. Knockdown or overexpression efficiency in HuCCT1 or RBE cells**

HuCCT1 cells were transfected by NC- or 14-3-3ε-siRNA, while RBE cells were transfected by scrambled or pcDNA-3.1-14-3-3ε-Flag plasmid. Western blot analysis of the levels of 14-3-3ε or Flag.

**Fig. S3. Fifty most frequently altered neighbor factors around 14-3-3ε**

The protein-protein interaction with the 50 most frequently altered neighbor factors around 14-3-3ε generated by STRING database.

**Fig. S4. Effects of 14-3-3ε on PI-3K/Akt in HuCCT1 or RBE cells**

HuCCT1 cells were transfected by vector or 14-3-3ε-plasmid, while RBE cells were transfected by NC- or 14-3-3ε-siRNA. Western blot analysis of the expressions of p-PI-3K/p85 subunit and p-Akt.
